# Sclerostin's role in bone's adaptive response to mechanical loading

**DOI:** 10.1016/j.bone.2016.10.008

**Published:** 2017-03

**Authors:** Gabriel L Galea, Lance E Lanyon, Joanna S Price

**Affiliations:** a Newlife Birth Defects Research Centre, Institute of Child Health, University College London, London WC1N 1EH, United Kingdom; b School of Veterinary Sciences, University of Bristol, Langford House, Langford, Bristol BS40 5DU, United Kingdom

## Abstract

Mechanical loading is the primary functional determinant of bone mass and architecture, and osteocytes play a key role in translating mechanical signals into (re)modelling responses. Although the precise mechanisms remain unclear, Wnt signalling pathway components, and the anti-osteogenic canonical Wnt inhibitor Sost/sclerostin in particular, play an important role in regulating bone's adaptive response to loading. Increases in loading-engendered strains down-regulate osteocyte sclerostin expression, whereas reduced strains, as in disuse, are associated with increased sclerostin production and bone loss. However, while sclerostin up-regulation appears to be necessary for the loss of bone with disuse, the role of sclerostin in the osteogenic response to loading is more complex. While mice unable to down-regulate sclerostin do not gain bone with loading, Sost knockout mice have an enhanced osteogenic response to loading. The molecular mechanisms by which osteocytes sense and transduce loading-related stimuli into changes in sclerostin expression remain unclear but include several, potentially interlinked, signalling cascades involving periostin/integrin, prostaglandin, estrogen receptor, calcium/NO and Igf signalling. Deciphering the mechanisms by which changes in the mechanical environment regulate sclerostin production may lead to the development of therapeutic strategies that can reverse the skeletal structural deterioration characteristic of disuse and age-related osteoporosis and enhance bones' functional adaptation to loading. By enhancing the osteogenic potential of the context in which individual therapies such as sclerostin antibodies act it may become possible to both prevent and reverse the age-related skeletal structural deterioration characteristic of osteoporosis.

## Highlights

• Loading-related changes in osteocyte sclerostin expression spatially predict subsequent osteogenic responses.
• Acute sclerostin down-regulation is not sufficient for maximal osteogenic responses to loading.
• Inability to suppress sclerostin precludes bone gain following loading. Lack of sclerostin prevents bone loss in disuse.
• Sclerostin influences the osteogenic context in which loading acts as its deletion enhances functional adaptation.

## Graphical abstract

**Figure:**
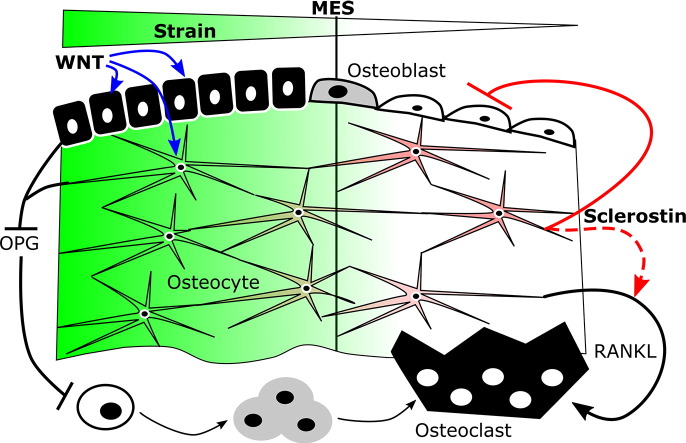

## 1 Introduction

Mechanical loading is the primary functional determinant of bone mass and architecture [1], [2]. Loading generates strain (percentage change in dimension) and other mechanically relevant stimuli (*e.g.* fluid flow shear stress) throughout the bone tissue and within the osteocyte canalicular network. Loading levels or distributions which engender strains beyond a habitual minimum effective strain (MES) trigger bone formation resulting in increased bone mass, improved bone architecture and thus re-establishment of habitual levels and distribution of strain [3], [4], [5]. Decreased loading, such as occurs during disuse, results in osteoclastic bone resorption and bone loss in an apparent attempt to also re-establish habitual levels and distribution of strain. This homeostatic feedback loop, described by Harold Frost as ‘the mechanostat’ [4], involves the site-specific co-ordinated (re)modelling activity of osteocytes, osteoblasts and osteoclasts [5].

Osteocytes are embedded in the mineralised matrix and were long thought to have little or no function, but are now known to play a particularly important role in coordinating local bone remodelling responses and have recently described as ‘master-regulators’ [6], [7], [8]. The Wnt antagonist *Sost*/sclerostin is almost exclusively expressed by osteocytes in the adult skeleton [9], and osteocytes are also an important source of receptor activator of nuclear factor κB ligand (Rankl) [10], which probably plays a key role in initiating repair in damaged bone; *e.g.* apoptosing osteocytes around microcracks secrete Rankl [11]. Canonical Wnt signalling in osteocytes also regulates bone resorption *via* the expression of osteoprotogerin (Opg); mice lacking β-catenin in osteocytes have dramatically reduced bone mass due to reduced Opg levels [12].

Given their location and morphology, with long interlinked dendritic processes forming a functional syncytium extending to the bone surfaces, osteocytes are ideally suited to sense load-associated strains, including shear strains across their membranes as fluid is displaced through their canalicular system. Osteocytes are now considered to be the primary mechanosensors which locally coordinate adaptive (re)modelling responses [13]. The experiment by Skerry et al. [2] that led us to acceptance of this hypothesis was the demonstration of rapid strain magnitude-related increases in the activity of the metabolism enzyme glucose-6-phosphate dehydrogenase (G6PD) in osteocytes in the turkey ulna following a short period of loading.

**Fig. 1:**
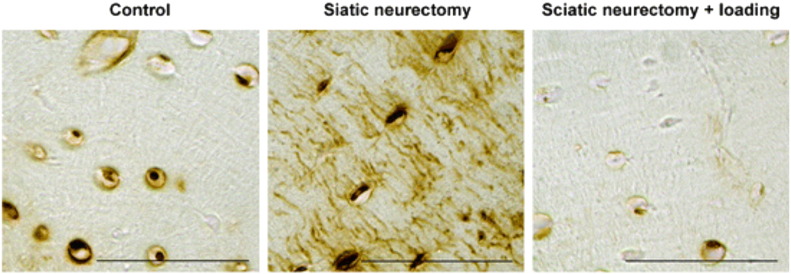
Mechanical loading decreases, whereas disuse increases sclerostin expression. Sclerostin immunolocalisation in tibial cortical bone osteocytes of control limbs subjected to normal cage activity, limbs subjected to disuse through sciatic neurectomy, and disused limbs subjected to exogenous osteogenic axial loading. Figure reproduced with permission from Moustafa et al. [19]. Scale bar = 50 μm.

**Fig. 2:**
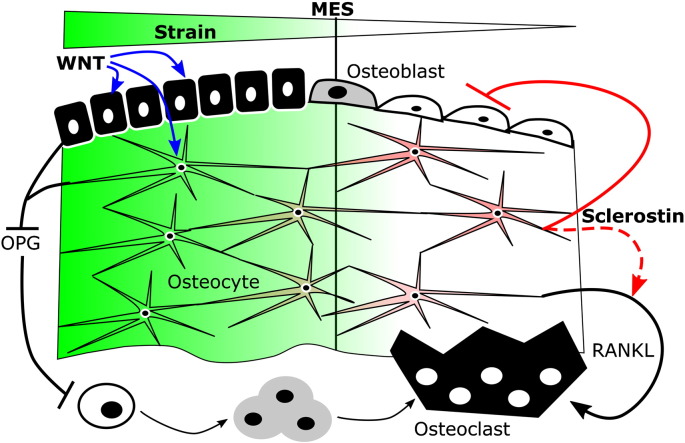
Simplified model describing sclerostin's roles in bones' adaptation to loading-engendered strains. Strains greater than the minimum effective strain (MES, green) are association with low osteoclast activity and increased osteoblast activity, whereas the low strains experienced in disuse are associated with reduced osteoblast activity and increased osteoclast activity. The activity of these effector cells is coordinated by osteocytes at least in part through sclerostin (red) secretion. In low strain conditions, sclerostin inhibits osteoblast function and may indirectly promote resorption through Rankl [73]. Strains greater than the MES down-regulate sclerostin, allowing activation of osteoblasts at least in part through canonical Wnt signalling, which may indirectly inhibit resorption through Opg expression.

For many years after this, the mechanisms underlying the coordination of adaptive remodelling responses by osteocytes were largely unknown. Hypothesised mechanisms included direct cell-cell communication [14], [15] and/or the secretion of paracrine mediators such as prostaglandins (PG) or insulin-like growth factors (Igf). However, once sclerostin had been shown to be expressed in osteocytes [9], Robling et al. [16] convincingly demonstrated that one potentially important mechanism by which mechanical loading controls osteocyte activity is by regulating sclerostin expression. His demonstration that loading the mouse ulna down regulates sclerostin expression has been reproduced in a variety of experimental loading models [17], [18], [19], [20], [21], [22], [23] (Fig. 1). It then led to proposal of the simple model that local, loading-related down-regulation of osteocyte sclerostin increases bone formation by relieving inhibition of canonical Wnt signalling in osteoblasts while also, directly or indirectly through regulation of OPG, suppressing the resorptive activity of osteoclasts (Fig. 2). The responses of transgenic mice with altered sclerostin expression to changes in loading strongly support the validity of this model. However, recent findings of sclerostin-independent changes in bone formation following loading [24] have demonstrated that this model is somewhat over-simplified. Furthermore, the mechanisms by which loading-related stimuli initiate this process by down-regulating sclerostin have only been partially explored.

## 2 Loading-related changes in bone mass reflect sclerostin regulation

The model presented in Fig. 2 is largely based on the demonstration that the cross-sectional distribution of strains engendered within loading-responsive regions of mouse long bones spatially parallel the acute down-regulation of sclerostin protein (within 24 h following an episode of loading [18]) and subsequent increases in bone formation. As described above, this was first demonstrated in the mouse ulna subjected to non-invasive axial loading [16]. Axial loading of the mouse ulna generates different magnitudes of mechanical strain in the bone's proximal, middle and distal regions as well as in different cross-sectional sectors at the same longitudinal site. Strain magnitudes were found to correlate with both the increase in bone formation and the down-regulation of sclerostin within these regions. Conversely, the reduction in strain experienced through tail suspension-induced disuse increased *Sost* RNA expression in the mouse tibia. However, protein level analysis of sclerostin expression by immunohistochemistry following tail suspension did not detect changes in the proportion of osteocytes stained positive for sclerostin around the level of the tibia/fibula junction [16].

The lack of change in sclerostin expression around the mouse tibia/fibula junction during tail suspension is potentially consistent with the finding that this region appears to be the least affected by disuse, with the most significant bone loss occurring proximal and distal to this region [25]. In a later study, Moustafa et al. [19] mapped site-specific changes in sclerostin expression in the mouse tibia using immunohistochemistry following unilateral axial loading. In cross-sections from the highly load-responsive proximal tibia, the increase in bone formation and decrease in osteocyte sclerostin expression correlated with the mechanical strains predicted by finite element model analysis. In contrast, in the distal tibia below the tibia/fibula junction, sclerostin was not down-regulated and bone formation did not increase following loading. In the same study, disuse following sciatic neurectomy increased sclerostin expression in both the proximal and distal tibia, and additional loading after disuse significantly reduced sclerostin expression in both sites, although the magnitude of the effect was greater proximally. Similar site specificity was also observed in the trabecular compartment of the proximal tibia: loading reduced sclerostin expression and increased bone gain in the secondary spongiosa, but in the primary spongiosa no bone formation was observed nor any associated down regulation of sclerostin expression. These detailed analyses demonstrate that the spatial distribution of bone loss with disuse and of bone formation following loading closely follow the early changes in sclerostin expression. However, none of the studies published to date correlating changes in sclerostin expression with the spatial distribution of bone formation flowing loading have shown that the two are causally related. The relationship between sclerostin regulation and bone (re)modelling is clearly complex as both continuous (catabolic) and intermittent (anabolic) parathyroid hormone (PTH) treatments down-regulate *Sost* despite having opposite effects on bone mass [26], [27], [28].

Evidence that the spatial correlation between loading-related sclerostin regulation and changes in bone (re)modelling may be causal is provided by loading studies using different genetically modified mouse models. Sclerostin knockout mice do not show bone loss in response to disuse induced by hind limb unloading [29] or botulinum toxin injection [24], suggesting that sclerostin up-regulation is necessary for disuse-induced bone loss. To determine whether sclerostin down-regulation following increased loading is necessary for subsequent bone formation, transgenic mice harbouring the human SOST gene driven by an 8 Kb Dmp1 promoter (Sost^Tg^) were generated [20]. Ulna axial loading down-regulates endogenous, but not human, *Sost* expression in these mice. Further supporting evidence that sclerostin down-regulation is required for loading-induced bone formation, was the observation that loading induced significantly greater bone formation in wild type than Sost^Tg^ mice. These independent studies specifically test the roles of sclerostin in bone's adaptation to loading and as such provide strong evidence that both loading-related bone gain and disuse-associated bone loss require changes in sclerostin expression, at least in young mice.

Evidence supporting the potential importance of *Sost* down-regulation in bones' osteogenic response to loading also comes from studies utilising mice with genetic modifications in mechano-responsive pathways which result in altered *Sost* regulation following loading. For example, increased basal sclerostin expression, abrogation of sclerostin down-regulation with loading and reduced load-related bone formation is observed in periostin knockout (Postn^−/−^) mice [22]. Similarly, four point tibial bending of mice lacking osteocytic Igf1 expression does not result in *Sost* down-regulation and triggers a diminished osteogenic response to loading compared with wild type controls [23]. In contrast, deletion of the androgen receptor in male androgen receptor (AR) knockout mice is associated with greater sclerostin down-regulation and enhanced bone formation following loading compared with wild type controls [21]. Taken together, these studies provide examples of situations in which changes in sclerostin regulation are associated with altered adaptive responses to loading.

## 3 Mechanisms underlying sclerostin down-regulation by loading

**Fig. 3:**
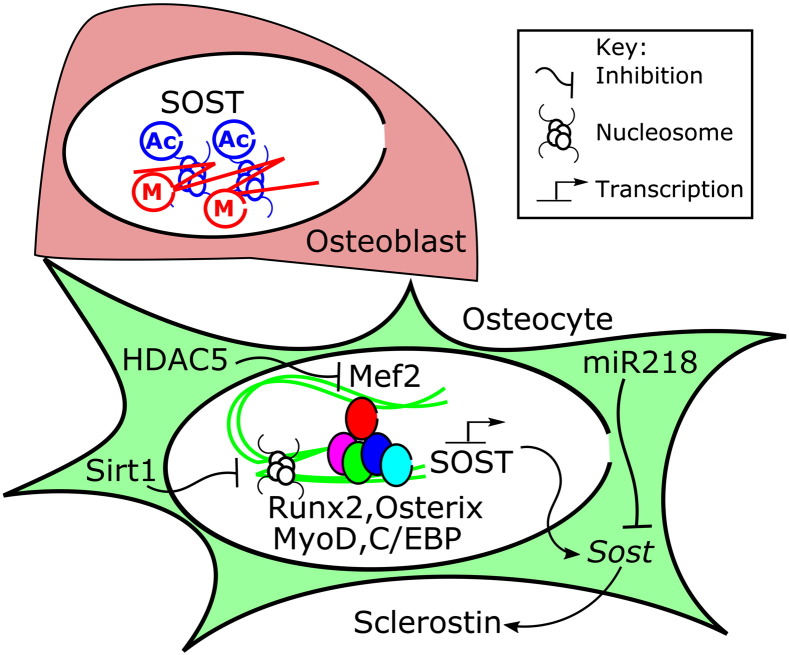
Simplified schematic representation of transcriptional and epigenetic regulators of basal *Sost* expression. In osteoblasts, the SOST gene is epigenetically repressed through DNA methylation (M) and potentially histone acetylation (Ac). HDACs also fine tune SOST promoter and Mef2-dependant enhancer activity in cells which express *Sost*. Transcriptional regulators able to bind the SOST promoter include osteoblast-specific (Runx2, osterix) and non-bone-specific (MyoD, C/EBP) transcription factors. Once expressed, Sost RNA stability is influenced by micro-RNAs including miR218.

The above *in vivo* studies describing altered basal sclerostin expression and changes in the load-related regulation of sclerostin in genetically modified mice, while informative, provide limited insight into the molecular mechanisms by which osteocytes regulate sclerostin expression. Instead *in vitro* studies using a variety of model systems have been required to address this. These studies have shown that the basal rate of sclerostin expression is under both transcriptional and broader epigenetic control (Fig. 3). Its restricted expression in osteocytes is achieved through an epigenetic mechanism; the SOST promoter is DNA methylated in osteoblasts but becomes demethylated during the osteoblast to osteocyte transition, allowing initiation of gene expression [30]. Transcription factors known to bind elements in the demethylated SOST promoter include the bone-specific transcription factors Runx2 and Osterix [31], [32]. Bone non-specific transcription factors such as MyoD and C/EBP also bind the SOST promoter in human Saos-2 cells [31]. The ability of these various factors to regulate *Sost* expression is epigenetically determined by histone deacetylase (HDAC) enzymes such as Sirt1 and HDAC5 [33], [34], and once expressed *Sost* RNA stability is influenced by micro-RNAs such as miR-218 [35].

SOST promoter activity is enhanced by Mef2 binding to a distal enhancer element and inhibition of this binding is one of the mechanisms by which *Sost* is down-regulated by PTH [34], [36], [37]. Similar mechanistic studies into *Sost* regulation by strain have been hindered by the limited availability of cellular models. Primary osteoblasts do not express readily detectable levels of *Sost* until they form mineralised matrix, which precludes their use for *in vitro* strain studies. Mouse osteocytic MLO cell lines do not reliably produce readily detectable levels of *Sost* [38] and their expression of the constitutively active SV40 antigen [39] impacts PI3K/AKT signalling, which is a stain-responsive pathway [40]. The more recently developed IDG-SW3 cell line promises to circumvent this limitation, but these cells only express *Sost* after prolonged periods of differentiation [41]. Not surprisingly few osteoblastic cell lines express detectable *Sost*. However, rat UMR-106 osteosarcoma cells do respond to strain [40] and express very high levels of *Sost* in a manner akin to them having a constitutively active gene [38], but this makes the physiological relevance of this model questionable. In contrast, human Saos-2 osteosarcoma cells are also mechanoresponsive, but only confluent cultures express readily detectable *Sost* RNA and sclerostin protein [41], [42], [43] which is why we have used this model system. Subjecting subconfluent cultures of Saos-2 cells to *in vitro* strain by four point bending increases their proliferation [43], [44], whereas confluent cultures up-regulate osteocalcin and down-regulate *Sost* over a time course which parallels that seen in rodent bones following *in vivo* mechanical loading [43], [45].

Using the Saos-2 model we initially reported that *Sost* down-regulation by strain involves Cox2-initiated PGE2 signalling through an EP4/ERK pathway [45], consistent with a previous report that selective treatment with an EP4 agonist enhances the osteogenic responses to mechanical loading *in vivo* [46]. The importance of this pathway in the mechanical regulation of *Sost* expression is further demonstrated by the recent report that Cox inhibition with carprofen prevents sclerostin down-regulation in the ulnae of mice subjected to axial loading [47]. Cox2 upregulation in mechanically-stimulated osteoblastic cells is abrogated by inhibition of nitric oxide (NO)/protein kinase G (PKG) signalling down-stream of calcium signalling [48]. Inhibition of the NO synthase (Nos) enzyme also abrogates fluid shear-induced *Sost* down-regulation in osteoblastic cells [49], whereas long bone derived osteoblastic cells from AR knockout mice, which show enhanced sclerostin down-regulation *in vivo*, produced higher levels of NO when subjected to fluid shear *in vitro* [21].

AR, NO and PGE2 signalling pathways are all influenced by estrogen receptors (ERs), which also interact with canonical Wnt pathway components in mechanically strained osteoblastic cells [50], [51]. Our group and others have shown that the ERs, particularly ERα, are mediators of bone's adaption to loading (as reviewed in [50]). Global deletion of ERα greatly diminishes cortical osteogenic responses to loading [52] thus we were surprised to observe that blockade of ERα does not prevent *Sost* down-regulation by strain in Saos-2 cells, rather ERα inhibition *in vitro* or global deletion *in vivo* reduces basal *Sost* levels [43]. However, this observation is consistent with the subsequent demonstration that deletion of ERα in mature osteoblasts and osteocytes does not impair the adaptive response to axial tibial loading in female mice [53], [54]. In contrast, ERβ blockade does not alter basal *Sost* levels, but prevents strain-induced *Sost* down-regulation in Saos-2 cells [43]. Although the role of ERβ in bone's adaptation to loading has not been extensively investigated, it is worth noting that ERβ enhances Cox2 up-regulation [55] and ERK activation [56] following mechanical stimulation in different *in vitro* models.

Both these roles of ERβ are consistent with a down-stream Cox-2/PGE2/ERK pathway mediating *Sost* down-regulation following strain, although ERβ may also act down-stream of PGE2 signalling as PGE2 treatment increases estrogen response element activation in osteoblastic cells [57]. Interestingly ERβ knockdown prevents periostin up-regulation by estradiol in periodontal ligament cells [58] and given periostin knockout mice do not show significant sclerostin down-regulation [22], ERβ's role in sclerostin regulation may be through periostin as well as through ERK activation. Activation of ERK could be either up-stream of periostin action and/or down-stream of its binding to integrin receptors, including integrin α_V_ [3], [59], [60] and deletion of integrin α_V_ in the osteoblast lineage prevents *Sost* down-regulation in the ulnae of mice subjected to axial loading [61]. Integrin α_V_ directly interacts with and facilitates Igf1/Igf1R signalling [62], [63], which is potentially consistent with the report that osteocyte Igf1 deletion also abrogates loading-induced *Sost* down-regulation [23]. Intriguingly, integrin α_V_ also facilitates opening of connexin (Cx)43 hemichannels and Cx43 facilitates the release of PGE2, which is involved in the rapid activation of β-catenin in osteoblastic cells subjected to mechanical stimulation *in vitro* [64], [65]. However, integrin α_V_ expression is not required for ERK activation in calvarial osteoblastic cells subjected to fluid shear [61].

**Fig. 4:**
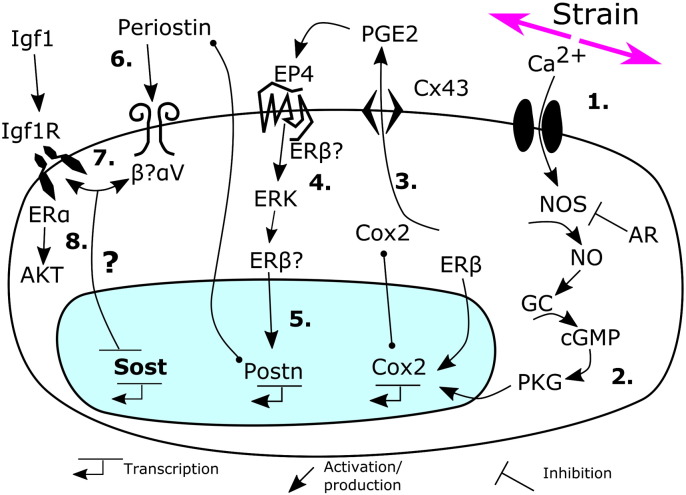
Schematic representation of pathways implicated in sclerostin down-regulation by mechanical stimulation. (1) Strain rapidly activates *Ca* signalling and down-stream (2) NO/guanylate cyclase (GC)/PKG signalling leading to up-regulation of Cox2, which also involves ERβ. Cox2 produces PGE2 (3) which is released at least in part through Cx43 hemichannels to activate EP receptors including EP4. EP4 activates ERK (4) signalling. Strain-induced ERK activation also involves ERβ and ER transcriptional activity is in turn increased in osteoblastic cells by PGE2. Activated ERβ can up-regulate periostin (Postn) expression (5). Periostin acts through integrins (6) including integrin α_V_, which interacts with the Igf1 receptor (7). Responses down-stream of Igf1R include ERα-mediated activation of AKT (8), however, the mechanisms by which the signalling cascades described inhibit Sost expression following exposure to strain remain unknown.

To date, no *in vivo* studies have been published that have systematically investigated the roles of different mechano-responsive signalling pathways in sclerostin regulation following loading. The majority of available studies are based on *in vitro* observations in osteoblastic cell lines subjected to defined mechanical stimuli which cannot fully replicate the effects of *in vivo* loading on the heterogeneous cell populations residing in and on bone. Currently, only Cox2/prostaglandin signalling has been demonstrated to acutely regulate sclerostin expression *in vitro*, suggesting a direct effect, and to also facilitate sclerostin down-regulation following loading *in vivo*. Furthermore, the mechanisms by which unloading results in sclerostin up-regulation have not been investigated and cannot be assumed to be the same as those which result in its down-regulation following increased loading. Nonetheless, putting the available jigsaw pieces together it is possible to propose a linear pathway which links early strain-related signalling events to ultimate down-regulation of *Sost* expression (Fig. 4). The sequence of events proposed in Fig. 4 is potentially consistent with the timing of gene expression changes seen following loading; *Cox*2 is up-regulated within 1–2 h [66] followed by *Postn* up-regulation around 6 h [22] and eventually *Sost* down-regulation 8–24 h after loading [47], [67]. However, the direct mechanisms by which loading-related stimuli decrease Sost promoter activity and/or reduce *Sost* RNA stability remain unknown and merit further study. The proposed model is also limited in assuming that all of the reported mediators of *Sost* down-regulation are involved in osteocytes' acute and immediate responses to strain. Bones' ability to respond to acute changes in loading is context dependent and multiple factors, local and systemic, are likely to influence the way *Sost* expression is regulated by loading; *e.g.* in a bone which has adapted its mass and architecture to the customary loads placed upon it, osteocytes and/or adjacent osteoblasts are likely to express factors which may limit or enhance strain-related *Sost* down-regulation.

## 4 Sclerostin itself influences the osteogenic context in which loading acts

Sclerostin itself is one such modulator of the osteogenic context; *e.g. in vitro*, its presence inhibits recruitment of Saos-2 cells to the cell cycle following mechanical strain or Wnt3a treatment, but not following treatment with estradiol [43], [44]. Sclerostin has also been shown to reduce proliferation and increase apoptosis in the absence of mechanical stimulation in other models [68], [69]. In addition, sclerostin has the potential to influence multiple signalling pathways that regulate various stages of the osteoblast lineage. Reported effects of sclerostin treatment on osteoblastic cells *in vitro* include inhibition of differentiation [70], [71], [72], inhibition of mineralisation [71], induction of RANKL expression [73], and promotion of osteocytic osteolysis [74]. Short term treatment of osteoblastic cells with recombinant sclerostin alters (predominantly down-regulates) the expression of a large number of genes, many of which are components of the Wnt signalling pathway [75]. This is consistent with sclerostin acting primarily as a canonical Wnt signalling inhibitor, although potential interactions with BMP and platelet derived growth factor (PDGF) cascades have also been reported [9], [72]. In the context of bone's response to loading, transgenic mice deficient for canonical Wnt co-receptors or the intra-cellular secondary signalling molecule β-catenin show diminished responses to mechanical loading [47], [76], [77]. β-Catenin is rapidly activated in osteocytes subjected to mechanical loading, but this response is diminished in osteocytes of mice unable to down-regulate sclerostin [20]. Taken together, these studies provide strong evidence that sclerostin acts as a canonical Wnt pathway inhibitor and that its down-regulation facilitates activation of this pathway following loading, but whether sclerostin directly or indirectly modulates other pathways following loading remains unknown.

## 5 Sclerostin down-regulation is not sufficient for load-related osteogenesis

The findings discussed thus far suggest that altered sclerostin expression is a critical osteocyte response to changes in mechanical loading and that sclerostin regulation permits/facilitates both adaptive osteogenesis when loads are increased and net resorption when they are decreased as in disuse. However, while it is clear that osteocytes, and sclerostin, are important for mediating bone's adaptive responses, it is wrong to assume that bone's responses to disuse and loading are regulated by the same mechanisms. This was suggested several years ago by a microarray study which showed that the genes and pathways regulated by loading are not all the same as those regulated by disuse [67]. Putting it another way; just because a cell or signalling pathway plays a critical role in the context of disuse, it does not mean that it will also be as important in regulating the bone formation response following loading. This is illustrated in an experiment which targeted ablation of osteocytes using diphtheria toxin [78]. Osteocyte ablated mice do not lose bone during unloading induced by tail suspension, however, osteocyte ablation does not prevent bone restoration caused by return to normal activity following a period of disuse. This suggests that either tail suspension induces bone loss through mechanisms unrelated to loading, such as increased glucocorticoid production [79], or that the responses of other cells to changes in loading are sufficient for normal bone gain following loading in the absence of osteocytes (and therefore sclerostin).

This latter interpretation is consistent with the recent report that Sost knockout mice do not lose bone due to unloading, but still show osteogenic responses to increased loading [24]. In fact, when loaded so as to generate equivalent strains, Sost^−/−^ mice show greater bone formation than wild-type controls. Thus, while viable osteocytes able to up-regulate sclerostin expression appear to be an absolute requirement for bone loss in disuse, down-regulation of sclerostin following loading does not appear to be so critical for the subsequent osteogenic response. That osteocytes are not the only cell involved in the adaptive response to loading should not come as a surprise given that numerous studies have shown that osteoblast-like cells are also mechano-sensitive. Well-established responses of osteoblast-like cells to strain include enhanced osteoblastic differentiation of marrow stromal cells (MSCs) as well as resumption of proliferation of cortical long bone derived osteoblastic cells [44], [52], [80], [81], [82]. Furthermore, the study which, to the authors' knowledge, was the first to demonstrate that osteocytes respond rapidly to changes in mechanical loading showed equally rapid responses (within 6 min) in adjacent periosteal cells [2].

The ability of osteoblasts to sense and respond to strain *in vitro* is clearly demonstrated by their ability to very rapidly enter into the cell cycle after strain exposure in the absence of sclerostin [18], [43], [44], [52]. *In vivo*, an increase in the number of osteoblasts on the periosteal surface is seen within 24 h following loading [18], although the location and nature of the proliferative osteoblast population remains undefined. A recent study on the effect of age on the loading response provides further evidence that down-regulation of sclerostin in osteocytes does not necessarily translate into an appropriate bone formation response. We hypothesised that in old mice loading would not down regulate sclerostin, but instead found that loading down-regulated sclerostin in 19-month-old mice to the same extent as in young (17-week-old) mice [18], even though the osteogenic response to non-invasive axial tibial loading was lower in old than in young animals. Interestingly this study showed that in old mice it was the ability of osteoblasts to proliferate that was compromised; osteoblast progression through the cell cycle following strain exposure *in vitro* and the increase in the number of periosteal osteoblasts following loading *in vivo* were impaired. These deficiencies in osteoblast function that occur with age may not only limit bone's adaptive responses to loading but also the beneficial effect of sclerostin neutralising therapies [83].

The finding that osteocytes in tibiae of old mice remain able to sense changes in mechanical loading and acutely respond by down-regulating *Sost* has recently been independently replicated by Holguin et al. [84]. In the Holguin study, a single bout of axial tibial loading effectively down-regulated *Sost* in 5-month-old as well as 12-month-old and 22-month-old mice, although the bone formation response was blunted with age. A possible explanation is that *Sost* RNA down-regulation is more transient in bones from 22-month-old than 5-month-old mice and others have shown changes in Wnt pathway-related gene transcripts and blunting of β-catenin activity in the old [84], [85], [86]. Intriguingly, Holguin et al. found that while repeated bouts of loading on subsequent days repeatedly down-regulate *Sost* in young mice, only the first bout of loading results in *Sost* down-regulation in the old. This suggests that old bone cells become refractory to repeated bouts of increased loading. However, we have recently reported that prior and concurrent disuse enhances the osteogenic response to repeated bouts of axial tibial loading in aged mice [87]. Whether this “rescue” of bone's response to loading in old mice is associated with the restoration of cells' ability to down-regulate sclerostin after each bout of loading needs to be determined. Nonetheless these studies demonstrate bone's “strain memory” influences subsequent responsiveness and that this relationship becomes less effective in the elderly. The relevance of these findings from rodent studies to elderly humans remains to be established.

## 6 Conclusions

Numerous studies have demonstrated that sclerostin plays a role in the effective working of the mechanisms associated with regulation of bone mass and architecture in relation to mechanical loading (the mechanostat). Sclerostin expression increases following unloading with the consequent inhibition of Wnt signalling and associated bone loss. Down-regulation of sclerostin is permissive for osteogenesis in response to loading, at least in part by relieving inhibition of canonical Wnt signalling. This is consistent with the potently osteogenic responses observed in humans treated with sclerostin-inhibiting antibodies now in advanced stages of clinical development [88]. However, sclerostin down-regulation in osteocytes is not the only process linking cellular mechanically related responses to functional remodelling as evidenced by mice lacking Sost having an enhanced response to loading. This is consistent with the emerging narrative that there is not a single linear pathway regulating bone's adaptive responses to loading, rather multiple pathways in which osteoblasts as well as osteocytes play important roles [50], [89], [90]. Elucidating the complex cellular mechanisms involved in mechano-responsiveness remains important because it could lead to the development of ‘smart’ novel therapeutic targets able to augment bones' specific physiological adaptive responses to loading-engendered stimuli rather than relying on non-specific, and largely ineffective, therapies to prevent or reverse loss of bone mass.
